# Risk Factors for Biochemical Recurrence After PSMA-PET-Guided Definitive Radiotherapy in Patients With *De Novo* Lymph Node-Positive Prostate Cancer

**DOI:** 10.3389/fonc.2022.898774

**Published:** 2022-06-07

**Authors:** Simon K.B. Spohn, Viktoria Birkenmaier, Juri Ruf, Michael Mix, August Sigle, Erik Haehl, Sonja Adebahr, Tanja Sprave, Eleni Gkika, Alexander Rühle, Nils H. Nicolay, Simon Kirste, Anca L. Grosu, Constantinos Zamboglou

**Affiliations:** ^1^ Department of Radiation Oncology, University Medical Center Freiburg, Faculty of Medicine, University of Freiburg, Freiburg, Germany; ^2^ German Cancer Consortium (DKTK), Partner Site Freiburg, German Cancer Research Center (DKFZ), Freiburg, Germany; ^3^ Berta-Ottenstein-Programme, Faculty of Medicine, University of Freiburg, Freiburg, Germany; ^4^ Department of Nuclear Medicine, University Medical Center Freiburg, Faculty of Medicine, University of Freiburg, Freiburg, Germany; ^5^ Department of Urology, University Medical Center Freiburg, Faculty of Medicine, University of Freiburg, Freiburg, Germany; ^6^ German Oncology Center, European University Cyprus, Limassol, Cyprus

**Keywords:** risk factors, PSMA-PET/CT, prostate cancer, radiotherapy, personalization, lymph node positive

## Abstract

**Introduction:**

The National Comprehensive Cancer Network recommends external beam radiotherapy (EBRT) combined with androgen deprivation therapy (ADT) as the preferred treatment option for newly diagnosed node-positive (cN1) prostate cancer (PCa) patients. However, implementation of positron emission tomography targeting prostate-specific membrane antigen (PSMA-PET) in the staging of primary PCa patients has a significant impact on RT treatment concepts. This study aims to evaluate outcomes and their respective risk factors on patients with PSMA-PET-based cN1 and/or cM1a PCa receiving primary RT and ADT.

**Methods:**

Forty-eight patients with cN0 and/or cM1a PCa staged by [^18^F]PSMA-1007-PET (*n* = 19) or [^68^Ga]PSMA-11-PET (*n* = 29) were retrospectively included. All patients received EBRT to the pelvis ± boost to positive nodes, followed by boost to the prostate. The impact of different PET-derived characteristics such as maximum standard uptake value (SUVmax) and number of PET-positive lymph nodes on biochemical recurrence-free survival (BRFS) (Phoenix criteria) and metastasis-free survival (MFS) was determined using Kaplan–Meier and Cox proportional hazard regression analyses.

**Results:**

Median follow-up was 24 months. Median initial serum prostate-specific antigen was 20.2 ng/ml (IQR 10.2–54.2). Most patients had cT stage ≥ 3 (63%) and ISUP grade ≥ 3 (85%). Median dose to the prostate, elective nodes, and PET-positive nodes was 75 Gy, 45 Gy, and 55 Gy, respectively. Ninety percent of patients received ADT with a median duration of 9 months (IQR 6–18). In univariate analysis, cM1a stage (*p* = 0.03), number of >2 pelvic nodes (*p* = 0.01), number of >1 abdominal node (*p* = 0.02), and SUVmax values ≥ median (8.1 g/ml for ^68^Ga-PSMA-11 and 7.9 g/ml for ^18^F-PSMA-1007) extracted from lymph nodes were significantly associated with unfavorable BRFS, but classical clinicopathological features were not. Number of >2 pelvic nodes (*n* = 0.03), number of >1 abdominal node (*p* = 0.03), and SUVmax values ≥ median extracted from lymph nodes were associated with unfavorable MFS. In multivariate analysis, number of >2 pelvic lymph nodes was significantly associated with unfavorable BRFS (HR 5.2, *p* = 0.01) and SUVmax values ≥ median extracted from lymph nodes had unfavorable MFS (HR 6.3, *p* = 0.02).

**Conclusion:**

More than 2 PET-positive pelvic lymph nodes are associated with unfavorable BRFS, and high SUVmax values are associated with unfavorable MFS. Thus, the number of PET-positive lymph nodes and the SUVmax value might be relevant prognosticators to identify patients with favorable outcomes.

## Introduction

Node-positive prostate cancer represents approximately 12% of *de novo* diagnosed prostate cancer (PCa) in the United States based on conventional imaging. Current National Comprehensive Cancer Network (NCCN v3.2022) guidelines recommend androgen deprivation therapy (ADT) with radiotherapy (RT) to the prostate and pelvic lymphatics in case of *de novo* pelvic lymph-node positive PCa (cN1 stage). ADT can be accompanied by abiraterone due to the recently reported benefit in metastasis-free survival (MFS) in this patient group in the STAMPEDE platform trial (1). In case of lymph node metastases above the aortic bifurcation (cM1a stage), current guidelines (NCCN v3.2022) recommend ADT with next-generation antiandrogens or doxetacel. In selected patients, RT to the prostate can be offered in addition to systemic therapy (2). Current risk-classification systems for these patient groups consider the localization of lymph node metastases (cN1 vs. cM1a stage). However, the number of affected lymph nodes and their biologic characteristics are not considered.

It is important to mention that all latter treatment recommendations and risk-classification systems are based on studies using conventional imaging for staging: computed tomography (CT), magnetic resonance imaging (MRI), and bone scintigraphy. The proPSMA study compared prostate-specific membrane antigen positron emission tomography (PSMA-PET) with conventional imaging for staging in high-risk PCa patients (3). Twenty-nine percent of patients undergoing first-line PSMA-PET were detected with pelvic nodal (20%) or abdominal nodal (9%) metastases (3). The authors depicted a significantly higher sensitivity and accuracy for PSMA-PET in the detection of lymph node metastases. Clinical trials correlating PSMA-PET-positive findings with histopathology from pelvic lymph node dissection in intermediate- to high-risk PCa patients showed somewhat poorer performance on a region level with a sensitivity between 40% and 66% and a specificity between 95% and 98% (4, 5). Several studies observed a significant impact of PSMA-PET imaging on RT treatment concepts in patients with primary PCa (6, 7). However, reports on the outcome after PSMA-PET-guided RT in PCa patients with *de novo* metastasized lymph nodes are sparse. Thus, we initiated this retrospective study in order to (i) evaluate the biochemical recurrence-free survival and (ii) its respective risk factors in a cohort of patients with initial cN1 and/or cM1a PCa receiving primary RT and ADT after PSMAPET staging.

## Patients and Methods

### Patients

This retrospective, mono-institutional analysis included patients with histologically proven PCa and cN1 and/or cM1a status in initial PSMA-PET/CT imaging. All patients received intensity-modulated RT (IMRT) with or without ADT from July 2015 to March 2021. The availability of PSMA-PET/CT scans at the maximum of 6 months prior to IMRT was mandatory. Patients were excluded from the analysis if not all PET-positive lymph nodes were included in the RT field or had cM1b/c disease (bone and/or visceral metastases) in PET. This study was approved by the institutional review board of the University of Freiburg (No.: 21-1149) and written informed consent was waived due to the retrospective character.

### PSMA-PET/CT Imaging

Radiolabeled tracers targeting the PSMA have been used for detection and delineation of lymph node metastases. PET/CT scans were performed 1 or 2 h after injection of the ligand ^68^Ga-PSMA-11 (*n* = 29) or ^18^F-PSMA-1007 (*n* = 19), respectively. The imaging systems used were GEMINI TF TOF 64, GEMINI TF 16 Big Bore, and Vereos (all from Philips, Netherlands). All imaging systems were cross-calibrated. A detailed description of our PSMA PET/CT imaging protocol is given in (8, 9).

### Treatment Protocol

Planning CT was acquired in supine position. Clinical target volume (CTV) and planning target volume (PTV) were created according to NRG and ACROP-ESTRO recommendations (10, 11). In all patients, the RT field comprised the prostate, the seminal vesicles, and the pelvic lymph nodes. In case of cN1 status, the upper border of the CTV was the aortic bifurcation. In case of cM1a status, the upper border of the CTV was 1 cm above the last PET-positive lymph node including elective pelvic nodes. Co-registered PET images were used to identify positive lymph nodes in the planning CT under consideration of the local nuclear medicine report and any SUV uptake higher than the background. PTV margin for boost volumes of PET-positive nodes was 1 cm. All patients received normo-fractionated IMRT and image-guided RT (IGRT). In the first step, RT was applied to the prostate, seminal vesicles, and elective lymphatics including a boost to PET-positive lymph nodes. In the second step, the prostate and the seminal vesicles received a sequential boost.

The aimed prescription dose was 76 Gy (EQD2, *α*/*β* = 1.6 Gy) to the entire prostatic gland and one-third of the seminal vesicles. No RT dose escalation to intraprostatic volumes was performed. Elective pelvic and abdominal lymphatics received 45–50.4 Gy in 1.8 Gy per fraction. Similar to (12), the aimed prescription dose for PET-positive lymph nodes was 54 Gy (EQD2, *α*/*β* = 1.6 Gy).

Administration of ADT was performed under consideration of patients’ individual preferences and comorbidities. Long-time ADT was strongly recommended to all patients concomitantly and adjuvant to EBRT. Neoadjvuant ADT was recommended if shrinkage of prostate volume was intended.

During follow-up (FU), patients were seen every 3–6 months for the first 2 years and every 6–12 months thereafter for physical examination and PSA measurements. FU examinations were performed at our institution or from another board-licensed urologist. Genitourinary (GU) and gastrointestinal (GI) toxicities were assessed according to common terminology criteria for adverse events (CTCAE) v5.0. Radiologic evaluation by PSMA-PET/CT was conducted in case of biochemical recurrence according to Phoenix criteria (13). In case of oligoprogression, metastasis-directed therapy (MDT) was offered to patients.

### Standardized-Uptake Value Analysis

All PET-positive lymph nodes were contoured by SS, applying validated contouring techniques (8, 9) under consideration of the local PET review and the anatomical borders on the corresponding CT scan in Eclipse planning treatment software 15.0 (Varian, USA). Subsequently, maximum standardized uptake values (SUVmax) were extracted from the prostate and lymph nodes.

### Statistical Analysis

The primary endpoint biochemical recurrence-free survival (BRFS) was defined as time from completion of IMRT until biochemical recurrent PCa according to the Phoenix criteria (13) or death from any cause. MFS was calculated from completion of RT until detection of any new metastases outside the RT field on PSMA-PET or death from any cause. Uni- and multivariate (forward logistic regression) Cox regression analyses were performed analyzing the impact of different clinical variables on BRFS. Therefore, variables were dichotomized: initial serum prostate-specific antigen (iPSA) ≤ and > 20 ng/ml, International Society for Urological Pathology (ISUP) grade < and ≥ 3, T-stage < and ≥ 3, cM1a stage, number of positive pelvic lymph nodes ≤ and > 2, and number of extrapelvic lymph nodes ≤ and > 1. SUVmax values within the prostate and within the PET-positive lymph nodes were dichotomized according to median values, respectively. To account for differences in SUVmax between ^68^Ga-PSMA-11 and ^18^F-PSMA-1007, the median was calculated for each tracer separately. For the graphical representation, the respective variables were analyzed by Kaplan–Meier survival curve compared by log-rank test. Mann–Whitney test and Wilcoxon matched-pairs signed rank test was used for *t*-test of unpaired and paired data. All tests were considered to be statistically significant at *p* < 0.05. Statistical analysis was conducted with SPSS v28 (IBM, USA). Descriptive statistics were performed with Excel 2016 (Microsoft Cooperation, USA) and GraphPad Prism v8.4.2 (GraphPad Software Inc, USA).

## Results

### Patient and Treatment Characteristics

Forty-eight patients with a median FU of 23 months (IQR 8–38 months) were included. See **Table 1** for patient characteristics. Twenty-nine patients underwent ^68^Ga-PSMA-11-PET and 19 patients underwent ^18^F-PSMA-1007-PET. cN1 stage was significantly different when considering CT scans only (*p* < 0.0001), whereas cM1a stage was not (*p* = 0.25). Median dose to the prostate, PET-positive lymph nodes, and elective nodes were 75 Gy, 55 Gy, and 45 Gy. Median SUVmax of the prostate and lymph nodes was 17.8 g/ml (IQR 9.9–28.9) and 8.1 g/ml (IQR 4.1–23.0) for ^68^Ga-PSMA-11 and 17.3 g/ml (IQR 9.3–31.9) and 7.9 g/ml (IQR 4.4–18.3) for ^18^F-PSMA-1007, respectively. Only one patient had PSMA-PET positive lymph nodes above the diaphragm. Cumulative acute grade 1 and 2 GI toxicities were 33% and 19%, and acute GU toxicities were 60% and 23%. Cumulative chronic grade 1 and 2 GI toxicities were 13% and 4%, and chronic GU toxicities were 13% and 6%. There was no significant difference of acute and chronic GU and GI toxicities between patients with and without cM1a stage (*p* > 0.11). No chronic grade 3 toxicities were observed.

**Table 1:** Patient characteristics.

Median age in years (range)	75 (58–86)
Median initial PSA in ng/ml (IQR)	20.2 (10.2–54.2)
ISUP grade	n (%)
1	0 (0)
2	5 (10)
3	16 (33)
4	12 (25)
5	13 (27)
n/a	2 (4)
cT stage	*n* (%)
1–2	12 (25)
3a	12 (25)
3b	18 (38)
4	6 (12)
cN1 stage according to PSMA-PET/CT	48 (100)
cN1 stage according to CT	32 (66)
cM1a stage according to PSMA-PET/CT	12 (25)
cM1a stage according to CT	10 (21)
Number of PSMA-PET positive pelvic lymph nodes	Median and IQR = 2 (1–4) *n* (%)
1	16 (33)
2	10 (21)
3	9 (19)
4	5 (10)
5	3 (6)
6	5 (10)
Number of PSMA-PET positive abdominal lymph nodes	*n* (%)
0	38 (79)
1	4 (11)
2	2 (4)
3	3 (6)
4	1 (2)
ADT	*n* (%)
Yes	43 (90)
No	5 (10)
Median duration of ADT in months (IQR)	9 (6–18)
Median PSA nadir (IQR)	0.17 (0.1–0.7)

PSA, prostate specific antigen; IQR, interquartile range; ISUP grade, International Society of Urologic Pathology grade; PSMA-PET, positron emission tomography targeting prostate-specific membrane antigen; ADT, androgen deprivation therapy.

During FU, 17 patients (35%) experienced a biochemical relapse. Eight patients were diagnosed with metastases in PSMA-PET/CT due to relapse, of which metastases were outside the RT field in 7 patients. Two-year and 4-year BRFS were 69% and 52%, respectively. Two-year and 4-year MFS were 75% and 52%, respectively.

**Figure 1:**
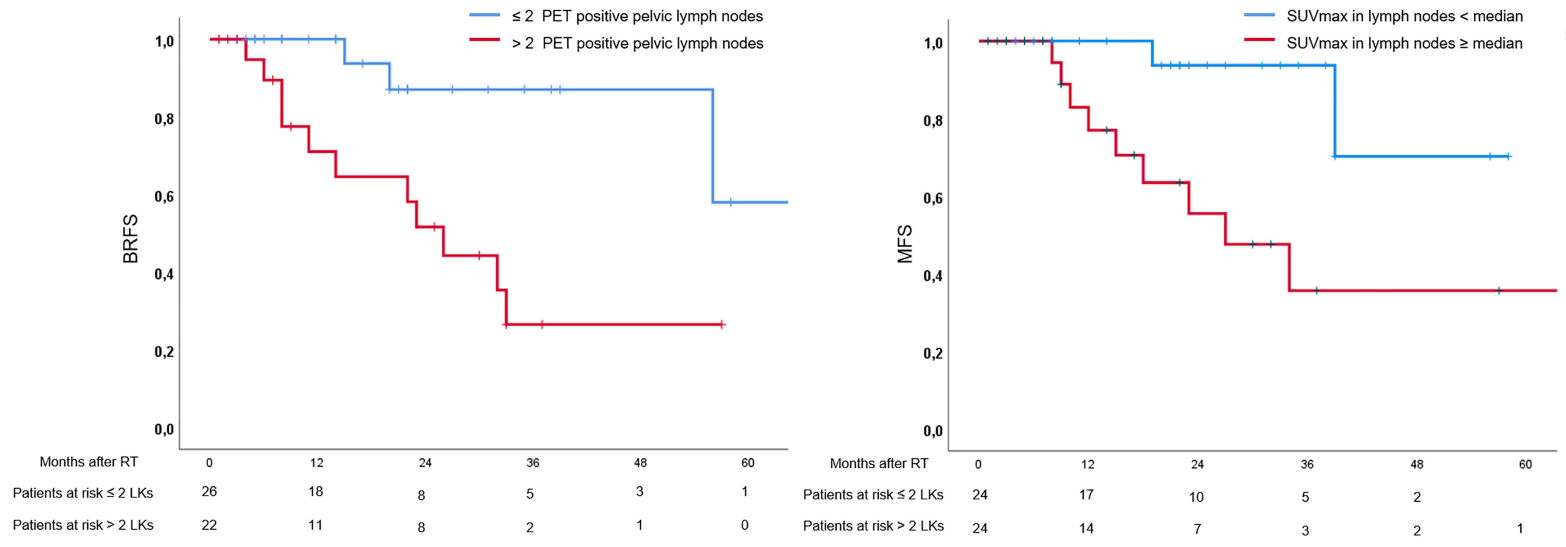
Kaplan–Meier curves. Left: Curves for biochemical recurrence-free survival in patients with > and ≤ 2 positive lymph nodes in positron emission tomography (PET). Right: Curves for metastasis-free survival (MFS) in patients with maximal standardized uptake values (SUVmax) ≥ and < median.

In univariate analysis, presence of cM1a stage (*p* = 0.03), >2 pelvic lymph nodes (*p* = 0.01), and >1 abdominal lymph node (*p* = 0.02) were associated with unfavorable BRFS, whereas the established clinical and pathological parameters were not (see **Table 2**). Additionally, SUVmax values ≥ median extracted from the prostate were not associated with BRFS (*p* = 0.89), while SUVmax values ≥ median extracted from lymph nodes were associated with unfavorable BRFS (*p* = 0.046). In multivariate analysis, only number of pelvic lymph nodes > 2 remained statistically significant (*p* = 0.01). In univariate analysis, presence of >2 pelvic lymph nodes (*p* = 0.03), >1 abdominal lymph node (*p* = 0.03), and SUVmax values ≥ median extracted from lymph nodes were associated with unfavorable MFS, while cM1a stage and clinicopathological parameters were not (see **Table 2**). In multivariate analysis, only SUVmax values ≥ median extracted from lymph nodes remained statistically significant regarding MFS (*p* = 0.02). See **Table 2** for details and **Figure 1** for Kaplan–Meier curves.

**Table 2:** Univariate and multivariate Cox regression. *p*-values and hazard ratio (HR) with 95% confidence interval (CI) are shown for biochemical recurrence-free survival (BRFS) and metastasis-free survival (MFS).

Univariate analysis	BRFS		MFS	
	*p*-value	HR (95% CI)	*p*-value	HR (95%CI)
Initial PSA	0.83	1.1 (0.4–3.2)	0.89	0.9 (0.3–3.0)
ISUP	0.94	1.1 (0.1–8.5)	0.69	0.7 (0.1–5.3)
cT stage	0.53	1.5 (0.4–5.4)	0.37	2.0 (0.4–9.5)
cM1a stage	**0.03**	3.7 (1.2–11.9)	0.24	2.3 (0.6–9.3)
>2 pelvic lymph nodes	**0.01**	5.2 (1.4–18.9)	**0.03**	5.7 (1.2–26.7)
>1 abdominal lymph node	**0.02**	4.3 (1.3–14.5)	**0.03**	4.6 (1.1–18.5)
SUVmax ≥ median in prostate	0.89	0.9 (0.3–2.6)	0.29	0.5 (0.1–1.8)
SUVmax ≥ median in lymph nodes	**0.046**	3.26 (1.02–10.4)	**0.02**	6.3 (1.4–29.2)
**Multivariate analysis**				
cM1a stage	ns			
>2 pelvic lymph nodes	**0.01**	5.2 (1.4–18.9)	ns	
>1 abdominal lymph node	ns		ns	
SUVmax ≥ median in lymph nodes	ns		**0.02**	6.3 (1.4–29.2)

PSA, prostate-specific antigen; ISUP, International Society of Urologic Pathology grade; cT stage, clinical tumor stage; cM1a, presence of extrapelvic lymph nodes; SUVmax, maximum standard uptake value; ns, non-significant. Statistical significant p-values are shown in bold.

## Discussion

To the best of our knowledge, this is one of the first studies to analyze treatment outcomes after RT in PCa patients with cN1 and/or cM1a status on initial PSMA-PET/CT. Despite prior clinical implementation of PSMA-PET, *de novo* lymph node-positive PCa has been scarcely investigated in clinical trials. Addition of RT to ADT has been proven to improve outcomes (14), but the utilization of PSMA-PET in primary staging leads to subsequent stage migration due to the increased detection rate of node and bone metastases. This, in addition to modern RT techniques, bears the potential to adjust and improve the management of node-positive PCa.

In our cohort, 4-year BRFS was worse than the results of a retrospective study (15) investigating moderately hypofractionated RT in node-positive PCa patients. All patients in this study received long-term ADT, and patients in our cohort had more advanced disease with >75% having cT3–4 stage, which might account for these differences. Despite a slightly different definition of BCR (namely, PSA > 1.5 ng/ml), results of the RTOG 8531 trial are in a more similar range with a 5-year BCFS of 54% in patients receiving RT + ADT (14). The recently published data from the STAMPEDE platform (1) demonstrate that outcomes can be significantly improved through intensification of systemic treatments by adding abiraterone to long-term ADT. Despite our cohort consisting of high-risk PCa patients with mainly cT3b and node-positive disease, duration of ADT was remarkably lower, which might be responsible for the poorer BRFS rates. Low rates of ADT might be attributed to patients’ preferences and comorbidities. However, improved outcomes through intensive systemic therapies come at the costs of adverse effects and financial burden. Consequently, further prognosticators are needed to identify patients who are at lower risk of relapse and thus would putatively benefit from intensified local therapies rather than systemic treatments. In this regard, our results demonstrate that RT achieves high local control rates, since only patients experienced in-field nodal recurrence.

In our analysis, established clinical and pathology parameters were not statically significantly associated with BRFS or MFS. This observation has also been previously described in studies investigating patients with PCa recurrence and reflects the putative clinical relevance of PSMA-PET-positive findings (16). These results suggest the hypothesis that patients with nodal spread diagnosed with PSMA-PET are at higher risk irrespective of the tumor extension and histopathology of the primary tumour. These established risk factors are particularly validated in localized PCa, but might be less relevant in patients with metastases. Presence of cM1a stage was statistically significant in univariate analysis, but only number of >2 positive pelvic lymph nodes remained significant in multivariate analysis. These results suggest that in the PSMA-PET era, the number of PET-positive pelvic lymph nodes might be a relevant prognosticator in patients with node-positive PCa undergoing RT + ADT. The prognostic role of the number of positive lymph nodes has been previously described by Briganti et al., who demonstrated that after radical prostatectomy and extended pelvic lymph node dissection, patients with ≤2 positive nodes experience improved cancer-specific survival compared to patients with >2 lymph nodes (17). Interestingly, we found the same cutoff for PSMA-PET-positive lymph nodes. Possibly, the historical differentiation between patients with pelvic and extrapelvic lymph node metastases might be less relevant than the tumor burden detected by PSMA-PET. Larger studies are warranted to confirm these results and investigate the role of extrapelvic nodal PSMA-positive spread. Nevertheless, these findings suggest that quantification of tumor burden is a potential tool to identify candidates who benefit from local treatments rather than from intensified systemic treatment. Local RT can be delivered to elective nodes or as part of an MDT. Elective nodal irradiation has been shown to improve outcomes compared to MDT in nodal oligorecurrence after surgery at the cost of toxicities (18). However, late toxicities were low in our cohort of patients who did not receive any prior treatment. Whether the optimal local treatment consists of irradiation of elective nodes or MDT in the setting of extended nodal spread needs to be assessed in future studies.

In addition to improved metastasis detection, PSMA-PET comprises biological information as it is using molecular tracers. It has been demonstrated that expression of PSMA correlates with worse GS and lymph node involvement in prostatectomy specimens and is associated with worse outcomes (19, 20). Since SUVmax correlates with PSMA expression (21), its analysis might enable to identity prognostic imaging biomarkers. We considered median SUVmax values for ^68^Ga-PSMA-11 and ^18^F-PSMA-1007 separately, since both tracers obtain different PSMA updates. In our exploratory analysis, SUVmax values ≥ median extracted from lymph nodes were associated with unfavorable BRFS, potentially representing patients with biologically more aggressive disease. Radiomic features (RFs) allow extracting deeper information from medical images (22), enabling non-invasive tumor characterization and prediction of lymph node involvement (23). Thus, image analysis through RF bears the potential to identify additional prognosticators and should be analyzed in the future. Interestingly, SUVmax values were the only significant parameter for MFS in multivariate analysis. These results suggest the hypothesis, that SUVmax might be a potential predictor to identify patients who are at higher risk for systemic progress, whereas patients with BRFS might suffer from in-field and out-of-field recurrence. The relatively low number of patients should be considered for interpretation of this statistical analysis. However, these interesting results need to be evaluated in larger cohorts with longer FU.

There are several limitations to our study. First, due to its retrospective character, PSMA-PET/CT, RT, and FU protocols were not consistent within all patients. Most notably, two different PSMA tracers were used. However, a study by Kuten et al. showed only small differences between both tracers by using histopathology as standard of reference (24). Second, no central review of PET imaging findings was performed. Thus, it is likely to have an ascertainment bias in the diagnosis of lymph nodes. Third, 5 patients in our study rejected the admission of ADT, and the median duration of ADT was only 9 months. Since it is unclear whether the inclusion of all PET-positive lesion into the RT field may allow a reduction of ADT, our study cohort had a possible undertreatment regarding systemic therapies. Finally, the FU time in our study is relatively short and the patient number is limited. Nevertheless, we believe that our study provides important results on RT of PET-positive lymph nodes, and the results should be evaluated in larger and preferably prospectively collected patient cohorts.

## Conclusion

Our results support the need for a more sophisticated differentiation of patients with *de novo* node-positive PCa. The number of PET-positive lymph nodes and the SUVmax value might be relevant prognosticators to identify patients with favorable outcomes.

## Data Availability Statement

The raw data supporting the conclusions of this article will be made available by the authors upon request.

## Ethics Statement

The studies involving human participants were reviewed and approved by the Institutional Review Board of the University of Freiburg. Written informed consent for participation was not required for this study in accordance with the national legislation and the institutional requirements.

## Author Contributions

SS and CZ contributed to the conception and design of the study. SS and VB collected data. SS concducted the statistical analysis. JR and MM were responsible for the conduction and reporting of the PSMA-PET/CTs. AS was involved in ADT treatment. EH, SA, TS, EG, AR, and SK were involved in radiotherapy treatment. NN and AG supervised the study. SS wrote the first draft of the manuscript. CZ wrote sections of the manuscript. All authors contributed to the article and approved the submitted version.

## Funding

This study was funded by the German Federal Ministry for Education and Research (BMBF JTC 2018 01KU1913) as part of the PersoProCaRisk grant.

## Conflict of Interest

The authors declare that the research was conducted in the absence of any commercial or financial relationships that could be construed as a potential conflict of interest.

## Publisher’s Note

All claims expressed in this article are solely those of the authors and do not necessarily represent those of their affiliated organizations, or those of the publisher, the editors and the reviewers. Any product that may be evaluated in this article, or claim that may be made by its manufacturer, is not guaranteed or endorsed by the publisher.

## References

[1] Attard G Murphy L Clarke NW Cross W Jones RJ Parker CC. Abiraterone Acetate and Prednisolone With or Without Enzalutamide for High-Risk non-Metastatic Prostate Cancer: A Meta-Analysis of Primary Results From Two Randomised Controlled Phase 3 Trials of the STAMPEDE Platform Protocol. Lancet (2022) 399(10323):447–60. doi: 10.1016/S0140-6736(21)02437-5 PMC8811484 34953525

[2] Parker CC James ND Brawley CD Clarke NW Hoyle AP Ali A. Radiotherapy to the Primary Tumour for Newly Diagnosed, Metastatic Prostate Cancer (STAMPEDE): A Randomised Controlled Phase 3 Trial. Lancet (2018) 392(10162):2353–66. doi: 10.1016/S0140-6736(18)32486-3 PMC6269599 30355464

[3] Hofman MS Lawrentschuk N Francis RJ Tang C Vela I Thomas P. Prostate-Specific Membrane Antigen PET-CT in Patients With High-Risk Prostate Cancer Before Curative-Intent Surgery or Radiotherapy (proPSMA): A Prospective, Randomised, Multicentre Study. Lancet (2020) 395(10231):1208–16. doi: 10.1016/S0140-6736(20)30314-7 32209449

[4] Hope TA Eiber M Armstrong WR Juarez R Murthy V Lawhn-Heath C. Diagnostic Accuracy of 68Ga-PSMA-11 PET for Pelvic Nodal Metastasis Detection Prior to Radical Prostatectomy and Pelvic Lymph Node Dissection: A Multicenter Prospective Phase 3 Imaging Trial. JAMA Oncol (2021) 7(11):1635–42. doi: 10.1001/jamaoncol.2021.3771 PMC8446902 34529005

[5] Gorin MA Rowe SP Patel HD Vidal I Mana-Ay M Javadi MS. Prostate Specific Membrane Antigen Targeted (18)F-DCFPyL Positron Emission Tomography/Computerized Tomography for the Preoperative Staging of High Risk Prostate Cancer: Results of a Prospective, Phase II, Single Center Study. J Urol (2018) 199(1):126–32. doi: 10.1016/j.juro.2017.07.070 PMC5802365 28736318

[6] Schmidt-Hegemann NS Eze C Li M Rogowski P Schaefer C Stief C. Impact of (68)Ga-PSMA PET/CT on the Radiotherapeutic Approach to Prostate Cancer in Comparison to CT: A Retrospective Analysis. J Nucl Med (2019) 60(7):963–70. doi: 10.2967/jnumed.118.220855 PMC6604695 30552203

[7] Sonni I Eiber M Fendler WP Alano RM Vangala SS Kishan AU. Impact of (68)Ga-PSMA-11 PET/CT on Staging and Management of Prostate Cancer Patients in Various Clinical Settings: A Prospective Single-Center Study. J Nucl Med (2020) 61(8):1153–60. doi: 10.2967/jnumed.119.237602 PMC7413232 31924715

[8] Spohn SKB Kramer M Kiefer S Bronsert P Sigle A Schultze-Seemann W. Comparison of Manual and Semi-Automatic [(18)F]PSMA-1007 PET Based Contouring Techniques for Intraprostatic Tumor Delineation in Patients With Primary Prostate Cancer and Validation With Histopathology as Standard of Reference. Front Oncol (2020) 10:600690. doi: 10.3389/fonc.2020.600690 33365271 PMC7750498

[9] Zamboglou C Fassbender TF Steffan L Schiller F Fechter T Carles M. Validation of Different PSMA-PET/CT-Based Contouring Techniques for Intraprostatic Tumor Definition Using Histopathology as Standard of Reference. Radiother Oncol (2019) 141:208–13. doi: 10.1016/j.radonc.2019.07.002 31431386

[10] Hall WA Paulson E Davis BJ Spratt DE Morgan TM Dearnaley D. NRG Oncology Updated International Consensus Atlas on Pelvic Lymph Node Volumes for Intact and Postoperative Prostate Cancer. Int J Radiat Oncol Biol Phys (2021) 109(1):174–85. doi: 10.1016/j.ijrobp.2020.08.034 PMC7736505 32861817

[11] Salembier C Villeirs G De Bari B Hoskin P Pieters BR Van Vulpen M. ESTRO ACROP Consensus Guideline on CT- and MRI-Based Target Volume Delineation for Primary Radiation Therapy of Localized Prostate Cancer. Radiother Oncol (2018) 127(1):49–61. doi: 10.1016/j.radonc.2018.01.014 29496279

[12] Jethwa KR Hellekson CD Evans JD Harmsen WS Wilhite TJ Whitaker TJ. 11c-Choline PET Guided Salvage Radiation Therapy for Isolated Pelvic and Paraortic Nodal Recurrence of Prostate Cancer After Radical Prostatectomy: Rationale and Early Genitourinary or Gastrointestinal Toxicities. Adv Radiat Oncol (2019) 4(4):659–67. doi: 10.1016/j.adro.2019.06.006 PMC6817538 31673659

[13] Roach M 3rd Hanks G Thames H Jr. Schellhammer P Shipley WU Sokol GH. Defining Biochemical Failure Following Radiotherapy With or Without Hormonal Therapy in Men With Clinically Localized Prostate Cancer: Recommendations of the RTOG-ASTRO Phoenix Consensus Conference. Int J Radiat Oncol Biol Phys (2006) 65(4):965–74. doi: 10.1016/j.ijrobp.2006.04.029 16798415

[14] Lawton CA Winter K Grignon D Pilepich MV. Androgen Suppression Plus Radiation Versus Radiation Alone for Patients With Stage D1/Pathologic Node-Positive Adenocarcinoma of the Prostate: Updated Results Based on National Prospective Randomized Trial Radiation Therapy Oncology Group 85-31. J Clin Oncol (2005) 23(4):800–7. doi: 10.1200/JCO.2005.08.141 15681524

[15] Mallick I Das A Arunsingh M. Moderately Hypofractionated Radiotherapy in Node-Positive Prostate Cancer. Clin Oncol (R Coll Radiol) (2019) 31(4):260–4. doi: 10.1016/j.clon.2019.01.004 30718087

[16] Emmett L Tang R Nandurkar R Hruby G Roach P Watts JA. 3-Year Freedom From Progression After (68)Ga-PSMA PET/CT-Triaged Management in Men With Biochemical Recurrence After Radical Prostatectomy: Results of a Prospective Multicenter Trial. J Nucl Med (2020) 61(6):866–72. doi: 10.2967/jnumed.119.235028 31676727

[17] Briganti A Karnes JR Da Pozzo LF Cozzarini C Gallina A Suardi N. Two Positive Nodes Represent a Significant Cut-Off Value for Cancer Specific Survival in Patients With Node Positive Prostate Cancer. A New Proposal Based on a Two-Institution Experience on 703 Consecutive N+ Patients Treated With Radical Prostatectomy, Extended Pelvic Lymph Node Dissection and Adjuvant Therapy. Eur Urol (2009) 55(2):261–70. doi: 10.1016/j.eururo.2008.09.043 18838212

[18] De Bleser E Tran PT Ost P. Radiotherapy as Metastasis-Directed Therapy for Oligometastatic Prostate Cancer. Curr Opin Urol (2017) 27(6):587–95. doi: 10.1097/MOU.0000000000000441 28816714

[19] Chu CE Alshalalfa M Sjöström M Zhao SG Liu Y Chou J. Prostate-Specific Membrane Antigen and Fluciclovine Transporter Genes Are Associated With Variable Clinical Features and Molecular Subtypes of Primary Prostate Cancer. Eur Urol (2021) 79(6):717–21. doi: 10.1016/j.eururo.2021.03.017 33840559

[20] Hupe MC Philippi C Roth D Kümpers C Ribbat-Idel J Becker F. Expression of Prostate-Specific Membrane Antigen (PSMA) on Biopsies Is an Independent Risk Stratifier of Prostate Cancer Patients at Time of Initial Diagnosis. Front Oncol (2018) 8:623. doi: 10.3389/fonc.2018.00623 30619757 PMC6307416

[21] Woythal N Arsenic R Kempkensteffen C Miller K Janssen JC Huang K. Immunohistochemical Validation of PSMA Expression Measured by (68)Ga-PSMA PET/CT in Primary Prostate Cancer. J Nucl Med (2018) 59(2):238–43. doi: 10.2967/jnumed.117.195172 28775203

[22] Spohn SKB Bettermann AS Bamberg F Benndorf M Mix M Nicolay NH. Radiomics in Prostate Cancer Imaging for a Personalized Treatment Approach - Current Aspects of Methodology and a Systematic Review on Validated Studies. Theranostics (2021) 11(16):8027–42. doi: 10.7150/thno.61207 PMC8315055 34335978

[23] Zamboglou C Carles M Fechter T Kiefer S Reichel K Fassbender TF. Radiomic Features From PSMA PET for non-Invasive Intraprostatic Tumor Discrimination and Characterization in Patients With Intermediate- and High-Risk Prostate Cancer - A Comparison Study With Histology Reference. Theranostics (2019) 9(9):2595–605. doi: 10.7150/thno.32376 PMC6525993 31131055

[24] Kuten J Fahoum I Savin Z Shamni O Gitstein G Hershkovitz D. Head-To-Head Comparison of (68)Ga-PSMA-11 With (18)F-PSMA-1007 PET/CT in Staging Prostate Cancer Using Histopathology and Immunohistochemical Analysis as a Reference Standard. J Nucl Med (2020) 61(4):527–32. doi: 10.2967/jnumed.119.234187 31562225

